# Sustained degradation of hyaluronic acid using an in situ forming implant

**DOI:** 10.1093/pnasnexus/pgac193

**Published:** 2022-09-17

**Authors:** Kelsey Hopkins, Kevin Buno, Natalie Romick, Antonio Carlos Freitas dos Santos, Samantha Tinsley, Elizabeth Wakelin, Jacqueline Kennedy, Michael Ladisch, Brittany L Allen-Petersen, Luis Solorio

**Affiliations:** Weldon School of Biomedical Engineering, Purdue University, West Lafayette, IN 47907, USA; Weldon School of Biomedical Engineering, Purdue University, West Lafayette, IN 47907, USA; Weldon School of Biomedical Engineering, Purdue University, West Lafayette, IN 47907, USA; Department of Agricultural and Biological Engineering, Purdue University, West Lafayette, IN 47907, USA; Laboratory of Renewable Resources Engineering, Purdue University, West Lafayette, IN 47907, USA; Department of Biological Sciences, Purdue University, West Lafayette, IN 47907, USA; Weldon School of Biomedical Engineering, Purdue University, West Lafayette, IN 47907, USA; Weldon School of Biomedical Engineering, Purdue University, West Lafayette, IN 47907, USA; Department of Agricultural and Biological Engineering, Purdue University, West Lafayette, IN 47907, USA; Laboratory of Renewable Resources Engineering, Purdue University, West Lafayette, IN 47907, USA; Department of Biological Sciences, Purdue University, West Lafayette, IN 47907, USA; Center for Cancer Research, Purdue University, West Lafayette, IN 47907, USA; Weldon School of Biomedical Engineering, Purdue University, West Lafayette, IN 47907, USA; Center for Cancer Research, Purdue University, West Lafayette, IN 47907, USA

**Keywords:** long acting injectable, protein/peptide, hyaluronidase, bioactivity, pancreatic cancer

## Abstract

In pancreatic cancer, excessive hyaluronic acid (HA) in the tumor microenvironment creates a viscous stroma, which reduces systemic drug transport into the tumor and correlates with poor patient prognosis. HA can be degraded through both enzymatic and nonenzymatic methods to improve mass transport properties. Here, we use an in situ forming implant to provide sustained degradation of HA directly at a local, targeted site. We formulated and characterized an implant capable of sustained release of hyaluronidase (HAase) using 15 kDa poly(lactic-co-glycolic) acid and bovine testicular HAase. The implant releases bioactive HAase to degrade the HA through enzymatic hydrolysis at early timepoints. In the first 24 h, 17.9% of the HAase is released, which can reduce the viscosity of a 10 mg/mL HA solution by 94.1% and deplete the HA content within primary human pancreatic tumor samples and ex vivo murine tumors. At later timepoints, as lower quantities of HAase are released (51.4% released in total over 21 d), the degradation of HA is supplemented by the acidic by-products that accumulate as a result of implant degradation. Acidic conditions degrade HA through nonenzymatic methods. This formulation has potential as an intratumoral injection to allow sustained degradation of HA at the pancreatic tumor site.

Significance Statement Hyaluronic acid (HA) is important in various biological functions and its quantity is normally balanced by HA synthase production and hyaluronidase degradation. However, sometimes this balance is not maintained leading to applications where it may be desirable to modulate HA exogenously. A prime example is pancreatic cancer where overabundant HA correlates with poor prognosis. The in situ forming implant described here provides sustained degradation of HA after a single injection, relying on the release of hyaluronidase and acidic degradation products. Thus, this implant has potential to be injected intratumorally to modulate the HA in the pancreatic tumor microenvironment. Modulating HA should improve mass transport into the tumors, providing a window of opportunity to deliver systemic chemotherapy with greater effectiveness.

## Introduction

Pancreatic cancer has the lowest 5-year survival rate of any major cancer at just 11% (1). Pancreatic ductal adenocarcinoma (PDAC) is the most common cancer of the pancreas with an estimated 60,000 new cases diagnosed per year in the United States (1). A hallmark of PDAC is the presence of desmoplasia, or dense stromal content. In fact, the stroma can account for as much as 90% of the tumor volume with the actual cancer cells occupying only the remaining 10% (2). This excessive stromal content in the pancreatic tumor microenvironment physically impedes drug delivery into the tumor and reduces the efficacy of systemically administered treatments (3–7). A major component of this desmoplasia is hyaluronic acid (HA) (8). HA is a megadalton glycosaminoglycan found naturally in the extracellular matrix (ECM), especially within the skin, joints, and vitreous humor of the eye (9, 10). HA is extremely hygroscopic, sequestering water and forming a viscoelastic gel that serves to aid in tissue lubrication and hydration as well as control the local fluid flow and interstitial transport (11). In PDAC, excessive HA creates a viscous stroma, which elevates the interstitial fluid pressure (IFP) collapsing vessel lumens and reducing drug transport into the tumor (12–14). Consistent with these findings, a high expression of HA correlates with poor prognosis and tumor aggressiveness (15, 16)

HA is a long, linear polysaccharide consisting of repeating disaccharide units of N-acetylglucosamine and glucuronic acid connected by $\beta $1,4 and $\beta $1,3 linkages (11, 17). There are different ways to degrade HA, including both enzymatic and nonenzymatic methods. Enzymatic degradation using hyaluronidases (HAase) is common and has been used clinically in a variety of applications (18–21). HAase works by catalyzing the hydrolysis of the $\beta $1,4 linkages, breaking the HA down into smaller fragments of variable length (17). Nonenzymatic methods include the degradation of HA using acidic conditions, alkaline conditions, ultrasonication, high temperatures, or the presence of oxidants/free radicals (22–24).

In the ECM of the skin, hyaluronan synthases produce HA, which is balanced by HAase degradation such that the turnover of HA is rapid, with a half-life of 24 h reported for HA in the skin (25). However, in PDAC this balance is dysfunctional, and HA significantly accumulates in the tumor resulting in a 12-fold increase relative to HA levels in the normal pancreas (26). Exogenous addition of HAase has been used within solid tumors to break down the HA barrier to mass transport, creating a window of opportunity for enhanced chemotherapy delivery into the tumor by reducing tumor IFP and swelling (27–30). However, the half-life of HAase is rather short at 2 to 3 min in plasma but can be extended to a half-life of 10.3 h with pegylation (i.e. PEGPH20) (27). In fact, treatment with PEGPH20 in combination with a chemotherapy agent in a PDAC mouse model was shown to deplete HA in tumors, significantly lower IFP, and restore vessel lumens leading to decreased tumor volumes and increased survival times (13) (30). However, PEGPH20 failed to produce results in a Phase III clinical trial (31). While there are any number of reasons for this result (32), we hypothesize that one contributing factor could be the systemic delivery of the hyaluronidase. Systemic drug delivery is transient, and the physical barriers present in the tumors would limit the amount of hyaluronidase that accumulates within the tumor, reducing the efficacy of the therapy. Thus, we used a type of long-acting injectable known as an in situ forming implant (ISFI), which can provide sustained, local drug release directly in the tumor (33–37). This system will allow for a more locally concentrated, continuous release of HAase, extending HAase's duration of action to better counteract the increased production of HA at the tumor site, without introducing systemic side effects.

ISFIs were first developed by Dunn et al. (38) and are currently used clinically for a range of applications (39–42). ISFIs are simple to manufacture and can be easily injected into the body via a small gauge needle (43). To form the implant, drug is mixed with a hydrophobic, biodegradable polymer dissolved in a water-miscible organic solvent (38). When this low-viscosity solution is injected into an aqueous environment, an exchange occurs between the solvent and the water present in the tissue that results in the formation of a solid drug-eluting depot from which drug is released for an extended period of time (44). Since the polymer is biodegradable, the implant does not need to be removed from the body, reducing the need for invasive surgical procedures. Of special note in relation to HA, as ISFIs degrade, acidic monomers are created that can drastically lower the pH surrounding the implant (45). Thus, acid-catalyzed degradation of HA can occur in addition to HAase release.

In this work, we developed an ISFI capable of sustained degradation of HA. Our approach with the ISFI was two-pronged. We formulated the implant to release bioactive HAase, which is especially effective at early timepoints, when both larger quantities of HAase are released and the HAase has yet to lose any significant bioactivity. Then, at later timepoints, as both the HAase release tapers off and the HAase has been subjected to the instability mechanisms present within the implants, we rely on the acidic byproducts created during implant degradation to lower the pH such that acid-catalyzed degradation of HA occurs. Therefore, degradation of HA occurs through both enzymatic and nonenzymatic degradation methods with our HAase-ISFI. Local, sustained release ensures prolonged exposure of the HA to these degradation mechanisms directly within the tumor site, which should create a better balance between HA synthesis and degradation in the tumor site.

## Results

### Release of hyaluronidase from implant

**Fig. 1.**
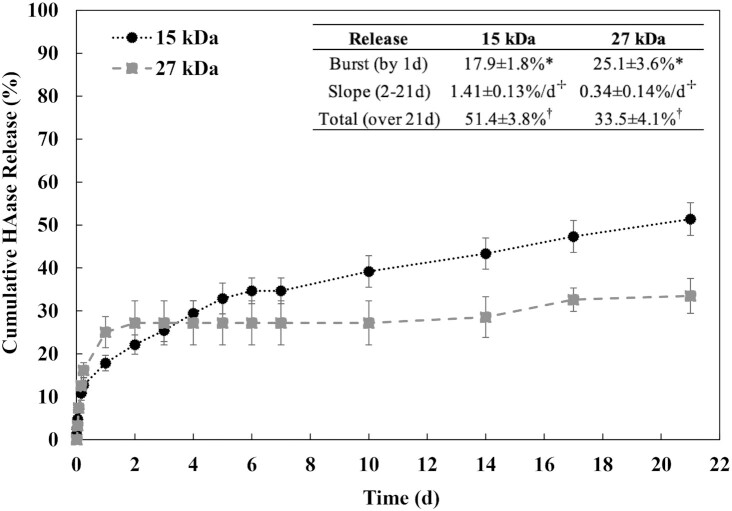
Cumulative release of hyaluronidase from the implant formulations. Release measured over 21 d with black circles indicating the 15 kDa implant and gray squares indicating the 27 kDa implant. (*n* = 3, error bars are mean ± SD). *, ⊹, † indicates a significant difference between 15 kDa and 27 kDa formulations (*P* <0.05).

Drug release from ISFIs can be tuned by altering different implant parameters, including the molecular weight of the polymer used to form the implant (46). Thus, in this study, two different implant formulations were created by using two different molecular weight PLGAs, 15 kDa, and 27 kDa. Fig. 1 shows the cumulative release of HAase from the implants over 21 d.

These molecular weights were selected to be below 27-kDa resulting in sufficiently low viscosities so that the polymers were injectable. The second characteristic sought for this polymer formulation was a semiconstant release profile of HAase from implant in tumor. For this purpose, PLGA was formulated with 15-kDa polymer, which corresponds to decreased burst and larger daily release compared to 27 kDa with increased burst and lower daily release. These polymers were selected as they provide a range of molecular weights that are injectable and have different daily release rates of HAase.

Both implants show a burst release of HAase within the first 24 h. The 15-kDa implant had a significantly lower burst release of 17.9% ± 1.8% of total enzyme released in 24 h compared to the 27-kDa implant with a burst release of 25.1% ± 3.6% in 24 h (*P* <0.05). Following the initial burst release, a more linear release phase was observed for the remainder of the study. However, the 15 kDa formulation had a significantly higher slope during this 2 to 21 d period [ 1.41% ± 0.13% of enzyme per day compared to 0.34% ± 0.14% per day (*P* <0.05)]. In total, after 21 d, the 15 kDa implant had cumulatively released significantly more enzyme than the 27-kDa implant (51.4% ± 3.8% vs. only 33.5% ± 4.1%, *P* <0.05). Because the 15-kDa formulation exhibited superior HAase release compared to the 27 kDa formulation as evidenced by increased slope and total enzyme release, the 15-kDa formulation was chosen moving forward for all future studies.

### Characterization of the implant delivery vehicle

**Fig. 2.**
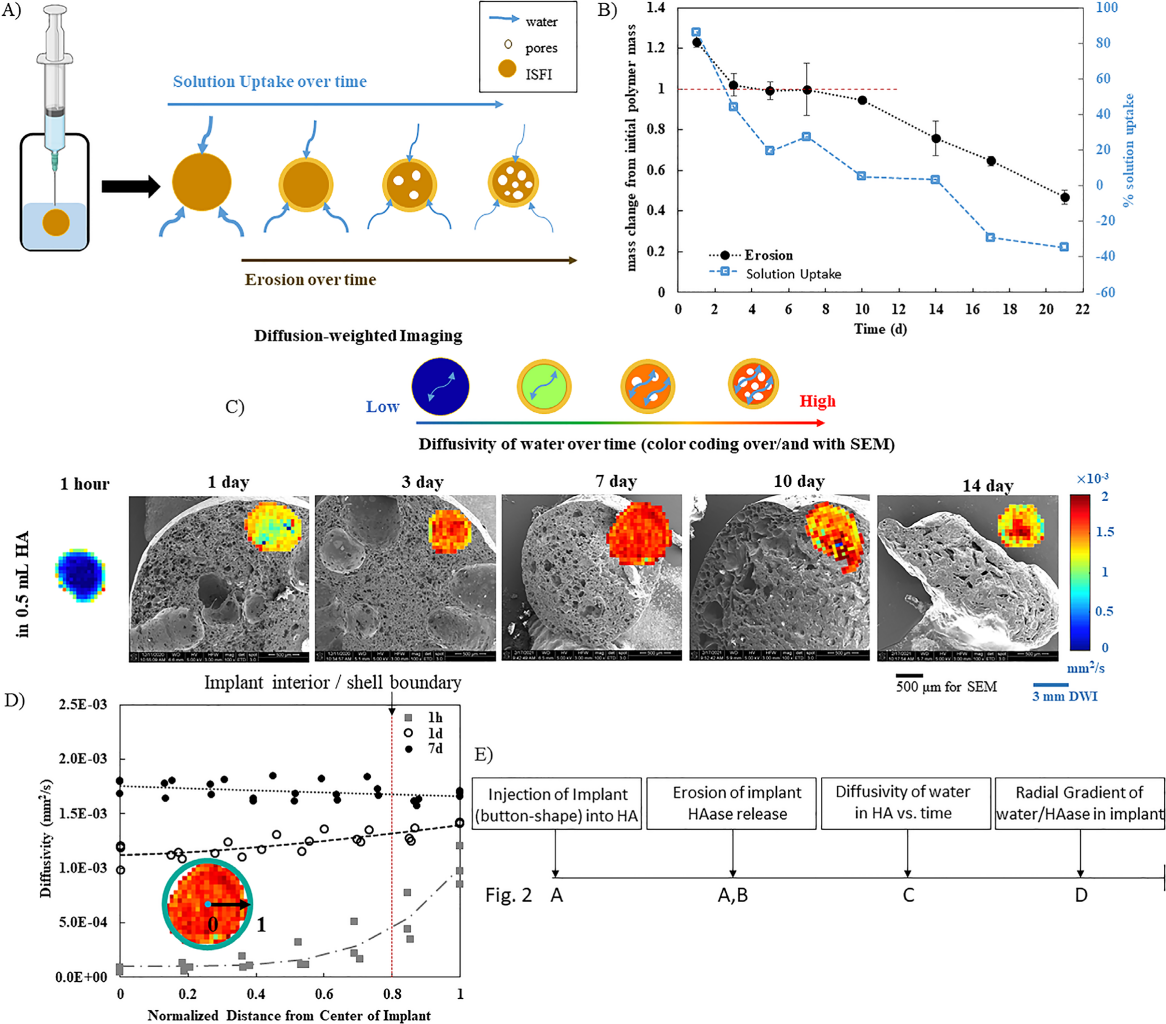
Characterization of the 15-kDa hyaluronidase implant delivery vehicle. (A) Schematic representation of injection of implant into HA, with implant erosion over time due to action of HAase and dissolution in aqueous phase over time. (B) Implant erosion and solution uptake. In black, erosion over 21 d normalized to the initial polymer mass; line at 1 represents the initial theoretical mass of polymer in the implant. In gray, percentage solution uptake of the implants over 21 d (for both: *n* = 3, error bars are mean ± SD). (C) Diffusion maps of water in the implant from diffusion-weighted imaging (DWI) overlaid on SEMs showing the implant microstructure. For DWI, red represents high diffusivity, while blue rerpresents low diffusivity. (D) Spatial profiles of diffusivity. Diffusivity of water in the implant measured as a function of distance from the center of the implant at selected timepoints. Line at a distance of 0.8 represents the border between the impant interior (0 to 0.8) and the implant shell (>0.8). (E) Schematic representation of sequence that represents monitoring of implant action and dissolution over the course of 14 to 21 d.

After choosing the 15-kDa formulation, implants were injected into 0.5 mL HA for characterization studies on the behavior of the implants themselves (Fig. 2A, cartoon of syringe).

#### Implant erosion and solution uptake

Fig. 2B shows the implant's erosion and solution uptake over 21 d. The onset of erosion begins when the implant's normalized residual mass drops below the 100% polymer line (red line in Fig.   2B) as this is when mass loss of polymer begins to occur. Residual solvent was still present in the implants through the first 7 d as the residual mass remained above 100%. Erosion then began between 7 and 10 d postformation (Fig. 2B). The residual mass dropped from 100% ± 13% at 7 d to 95% ± 1% at 10 d. The implants did not uptake bath solution over time, rather they had negative percentage solution uptake values at 21 d (−34.8% ± 6.1%), indicative of a lack of swelling.

#### Implant microstructure with SEM

SEM images were obtained from 1 to 14 d postformation (Fig. 2C). When injected into HA, these implants were uniformly microporous throughout, rather than having a clearly defined outer shell with large interior pores. These implants maintained this structure over time, although slightly decreasing in size by 14 d.

#### Diffusivity of water in the implant using DWI

Apparent diffusion coefficient (ADC) maps were used to visualize the diffusivity of water within the implant from DWI with each pixel representing a distinct diffusivity value in the implant. These values are on a color scale for ease of visualization with blue representing low diffusivity and red representing high diffusivity. Representative implant region of interest (ROIs) are shown in Fig. 2C, overlaid on the SEM images at the same timepoints. The full set of ADC maps can be found in Fig. S1. The interior of the implants started at a low diffusivity (blue) at 1 h as the implant initially consists of a more viscous polymer/solvent core with water only penetrating the exterior shell. As water penetrates further and solidifies the entire implant into an interconnected porous network, the diffusivity of the implants increased significantly from 1 h to 1 d by 7.8-fold. From 3 to 14 d, the diffusivity then remained constant at an average value of (1.59 ± 0.14)$\times {10}^{ - 3}$ mm^2^/s (Fig. S2).

More information was obtained by analyzing spatial profiles of the diffusivity in the implants. For selected timepoints, diffusivity was plotted as a function of normalized implant radius in order to visualize the full spatial profile (Fig. 2D). At 1 h, the spatial diffusivity profile shows low diffusivity throughout the implant interior rising rapidly to a maximum at the shell. After this initial timepoint, the implants then had flat spatial diffusivity profiles indicating a constant diffusivity throughout the entire implant rather than having a distinct diffusivity at the shell.

### Analysis of bioactivity and HA degradation in vitro

After fully characterizing the implant carrier material, we next assessed the bioactivity of the released enzyme, HAase, and looked at the ability of the implant to degrade HA.

**Fig. 3.**
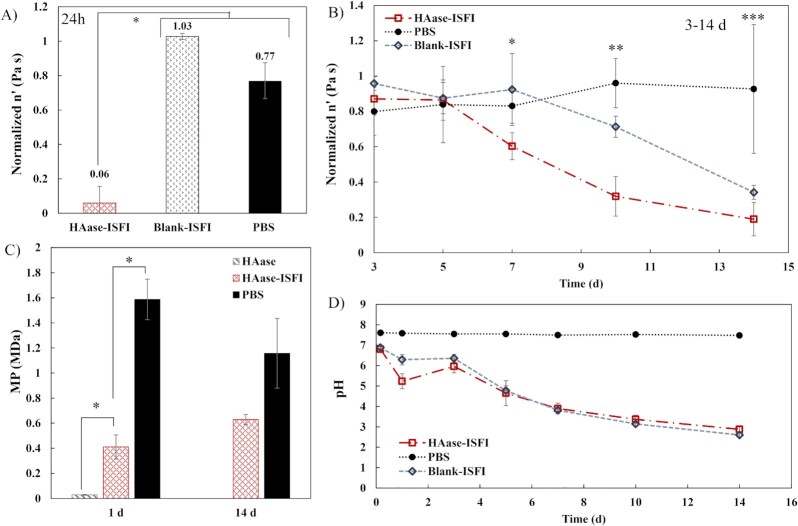
HAase functional testing on HA viscosity and molecular weight. (A) Viscosity of the HA solutions at 24 h normalized to the initial HA viscosity and (B) normalized viscosity of the HA solutions over the remaining 14 d comparing the effects of the hyaluronidase-releasing implant (HAase-ISFI) to a PBS control injection and a blank implant vehicle control (blank-ISFI). (C) Peak molecular weight (MP) of the HA solutions determined from size-exclusion chromatography (SEC) analysis. Timepoints (1 d and 14 d) were selected based on the viscosity results. (D) pH of the HA solutions at the same timepoints as the viscosity study for the same three groups. For all, asterisks indicate statistical significance (*P* <0.05) and error bars are mean ± SD. For (B): * = HAase-ISFI different from blank and PBS. ** = all three groups different from each other. *** = PBS control different from blank and HAase-ISFI (blank and HAase-ISFI not different from each other).

Changes in HA viscosity and molecular weight over 14 d were evaluated (Fig. 3). After 24 h of injection of a HAase-releasing implant, the viscosity of the HA was decreased to only 5.9% ± 9.6% of its initial value. This reduction in viscosity was significantly greater than that of both the phosphate buffered saline (PBS) control injection, which only slightly decreased the HA viscosity to 77.0% ± 10.4% of its initial value, and the injection of a blank (no drug) implant, which maintained the HA viscosity at 102.8% ± 1.8% of its initial value (*P* <0.05, Fig. 3A). The reduction in HA viscosity due to the HAase-implant was comparable to the reduction in HA viscosity after injection of a 2.5 mg/mL HAase positive control solution.

After HA bath solution replacement at 1 d and up to 5 d after the HAase-implant injection, the viscosity was not significantly different from the PBS control injection or the blank implant (vehicle control) (Fig. 3B). However, at 7 d, the HAase-implant had again significantly reduced the HA viscosity to 60.2% ± 7.6% of its initial value as compared to the PBS control (83.0% ± 9.8%) and the blank implant (92.4% ± 20.5%) (*P* <0.05). The HAase-ISFI continued to significantly reduce the HA viscosity at 10 d (31.8% ± 9.4% of initial) as compared to both the PBS control and the blank implant (*P* <0.05). However, at 10 d, the blank implant had also started to reduce the HA viscosity (71.3% ± 6.0% of initial), significantly lower than the PBS control. By the end of the 14 d study, the HAase-ISFI had reduced the HA viscosity to 19.0% ± 9.4% of its initial value, which was a significantly greater reduction than that of the PBS control, where the viscosity remained at 92.7% ± 36.5% of its initial value. However, by 14 d, the blank implant had also significantly reduced the HA viscosity to 34.1% ± 4.0% of its initial value, which was not significantly different from the HAase-ISFI. Thus, while the HAase-ISFI showed a significant effect by 7 d, the blank-ISFI took 10 d to show significant differences from the PBS control. Unlike for either of the implants, the HA viscosity of the PBS control samples did not change significantly over the entirety of the 14 d study.

To further understand the reduction in HA viscosity due to the blank implant, the pH of the HA solutions was measured at the same timepoints as the viscosity study (Fig. 3D). While the pH of the HA solution with the PBS control injection remained constant at 7.54 ± 0.05, there was a dramatic reduction in the pH of the HA solutions with either type of implant injected. The decline was especially evident after 5 d. By 14 d, the pH of the HA solution had dropped to 2.88 ± 0.17 (HAase-ISFI) and 2.60 ± 0.10 (blank-ISFI). We know that acidic (∼pH 3 or less) or basic (∼ pH 10 or more) solutions on their own can reduce the viscosity of HA (Fig. S3). Thus, we see the acidic by-products from implant degradation can significantly change the surrounding pH and lead to acid-catalyzed degradation of HA.

Samples of the HA solution used in the viscosity study were saved at selected timepoints for further analysis with SEC. SEC produced molecular weight distributions for each sample (Figs. S4 and S5). For the HA solutions of the PBS control injections, only one peak was seen on the spectra, which matched the expected profile of a 1.5 MDa HA solution. In contrast, the HA solutions of the implants exhibited a downward shift of this initial 1.5 MDa peak corresponding to a shift to lower molecular weight values. The peak molecular weight (MP) was used to quantify the molecular weight of that first peak. Fig. 3C shows the average MP values at 1 d and 14 d, timepoints chosen based on the viscosity study (Fig. 3A and B). At 1 d, the MP of the implant HA was 0.41 ± 0.10 MDa, which was significantly lower than the MP of the PBS control HA at 1.59 ± 0.16 MDa. The HA solutions of the 2.5 mg/mL HAase positive control injection exhibited a more significant downward shift in the molecular weight distribution with a 1 d MP of 0.03 ± 0.00 MDa. At 14 d, the downward spectra shift and reduction in MP due to the implant was still present although to a lesser, nonsignificant extent. The 14 d MPs were 0.63 ± 0.04 MDa for the implant and 1.16 ± 0.28 MDa for the PBS control.

### Analysis of bioactivity and HA degradation in ex-vivo tumors

#### Injection into murine pancreatic tumors

In order for these HAase-releasing implants to be most effective against pancreatic cancer, they would need to be injected into the pancreatic tumors to degrade the HA in the microenvironment. We wanted to test whether our implants could be injected into this dense stroma environment. Implants were injected into the tumors without incident, with the implant solution easily filling in the track created by the needle upon injection but not penetrating any further into the tumor beyond the needle track (Fig. S6). As a result, the implant shape mimicked the needle track, forming in a straight line across the length of the tumor cross-section, which can be seen in white in between the red arrows.

**Fig. 4.**
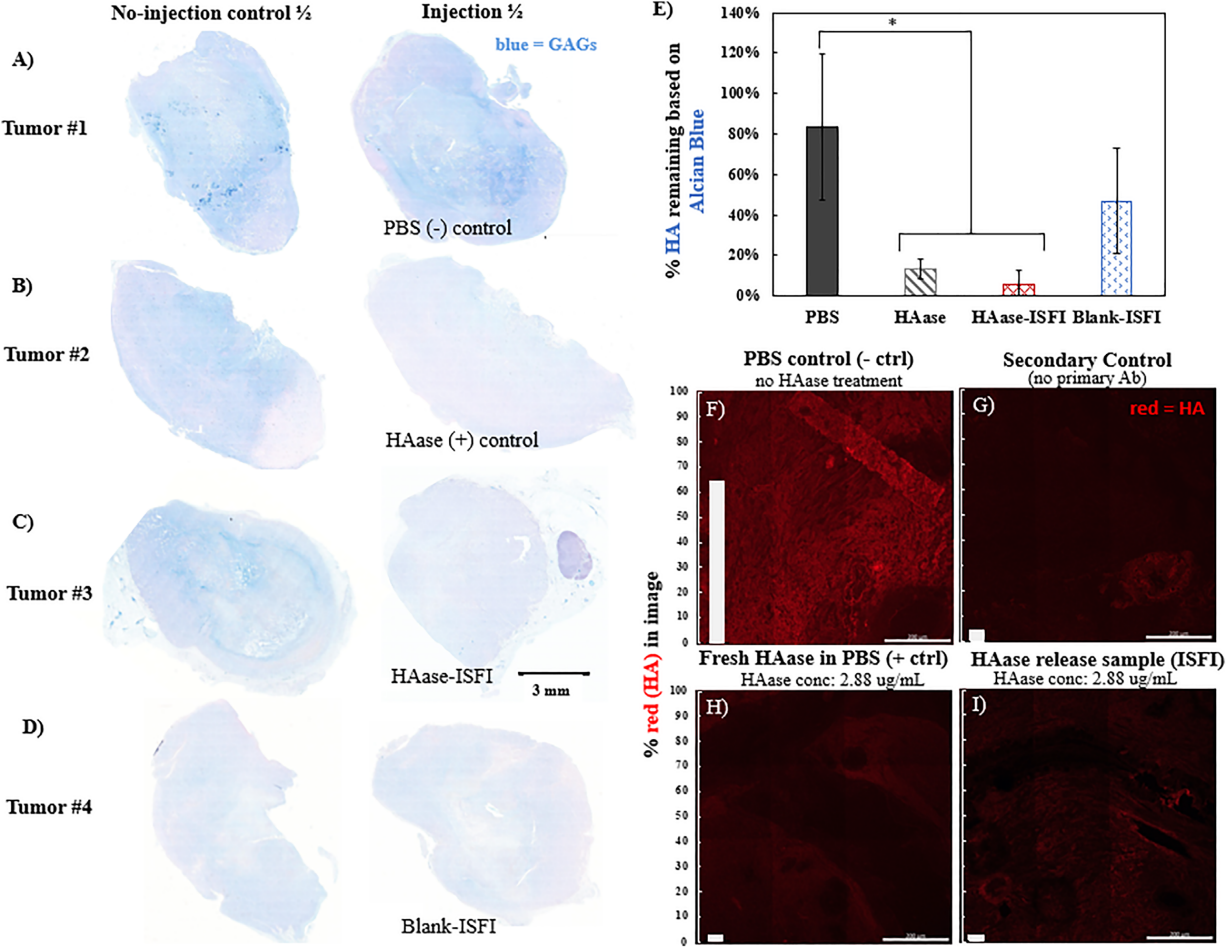
HAase functional testing ex vivo (A to E): Alcian blue pH 2.5 staining after injection into ex-vivo murine subcutaneous pancreatic tumors—one representative image from *n* = 3. For each tumor, one-half was left as a no-injection control and the other half was subjected to the following injections : (A) PBS, (B) 2.5 mg/mL HAase solution, (C) HAase-releasing ISFI, and (D) Blank implant. (E) shows the quantification of HA staining (blue), where * indicates significance *P* <0.05. (F to I): patient tumor slides were incubated at 37°C for 2 h with (F) PBS, (G) Secondary antibody control, (H) freshly-prepared HAase solution in PBS (2.88 µg/mL), and (I) implant release sample from the bath solution of a HAase-releasing implant at 1 d (HAase conc 2.88 µg/mL).

After injection of the implant and control solutions into the murine tumors, tumor slices were stained with Alcian blue pH 2.5 to assess glycosaminoglycan (GAG) content, which includes HA (Fig. 4). We saw abundant GAG content (blue) in the tumor injected with PBS that we associate with high levels of HA, and this staining greatly diminished after injection of a positive control HAase solution. Injection of the HAase-ISFI also greatly reduced the GAG staining after 2 d, indicating that our implant is releasing bioactive HAase in a 3D ex-vivo tumor environment. The change in alcian blue (HA) was then quantified after image deconvolution. The full set of images, including the hyaluronidase-treated serial slices, are shown in Fig. S7. Because each tumor had variability in the amount of HA within it, the percentage blue in the injection sample half was normalized to the percentage blue in the no-injection control half for each of the tumors. The results are shown in Fig. 4E. The PBS-injected tumor half still retained 83.6% ± 36.2% of the HA found in its corresponding no-injection tumor half (Fig. 4A). However, the 2.5 mg/mL HAase-injected tumor half significantly depleted this HA content, resulting in only 13.3% ± 5.0% of the original HA remaining (Fig. 4B). Similarly, the HA content in the tumor half injected with the HAase-ISFI was also significantly depleted, with only 5.9% ± 6.5% of the original HA remaining 2 d postinjection (Fig. 4C). Both the HAase positive control and the HAase-ISFI injections resulted in significantly lower HA quantities in the tumors compared to the PBS injection (*P* <0.05), and they were not significantly different from each other. A blank implant was also injected, which resulted in 46.8% ± 25.9% of the original HA remaining after 2 d (Fig. 4D).

#### Testing on human patient tumor samples

The ability of the released HAase to degrade HA content within serial sections of human PDAC tissue was used to evaluate the bioactivity of the released HAase on human HA. Substantial loss of HA (shown in red in Fig. 4F-I) was observed after incubating PDAC tissue with the HAase-implant releasate as seen by a reduction in the amount of red stain in Fig.   4I, resulting in only 2.0% of the image consisting of red (HA) pixels. In contrast, the PBS control showed abundant HA content (63.8% being red pixels, Fig. 4F), which was expected from tissue obtained from the pancreatic tumor microenvironment. The degradation of HA due to the implant releasate was comparable to the HA degradation observed with the HAase positive control solution at the same concentration (Fig. 4H). Finally, a secondary control was also done to show the nonspecific background staining that would result without the use of a primary antibody (Fig. 4G). The result was that the percentage of red (HA) in the image was 4.1%. The implant releasate solution and the HAase positive control solution both showed a loss of HA below this level, but a sample size of one did not allow for statistical comparisons.

### HAase kinetics

To estimate the rate at which the implant would need to release HAase in order to maintain constant enzyme activity levels in the tumor microenvironment, we estimated the HAase deactivation kinetics. Initially, there is a large burst release of HAase from the implants, but the HAase activity will decrease over time following first-order deactivation kinetics

(1) \begin{equation*} {\rm{A}}\left( {\rm{t}} \right){\rm{\ }} = {\rm{\ }}{A}_0{e}^{ - {k}_dt}, \end{equation*}

We solved for the deactivation rate constant, *k_d_*, by fitting Eqn. 1 to a HAase activity curve in PBS at 37°C (Fig. S8 and Tables S1 and S2). *k_d_* was calculated to be 0.11/d, corresponding to a half-life of 6.08 d in PBS. We then solved for the mass flow rate of HAase needed to maintain constant enzyme activity using (47):

(2) \begin{equation*} {\rm{E}}\left( {\rm{t}} \right){\rm{\ }} = {\rm{\ }}\frac{{{\mathit{ A}}_0}}{{{\mathit{ a}}_0}}{\mathit{ k}}_d, \end{equation*}

where *A*_0_ is the initial total activity (or the activity level we desire to maintain) and*a*_0_ is the initial specific activity of our batch of HAase (685 units/mg), resulting in an estimated mass flow rate of 25.0 µg/d of HAase from the implant needed to maintain the initial activity level of 150 units. Our 15-kDa implant formulation releases an average of 11.1 µg HAase/d. Thus, the implant release rate is on the same order of magnitude as the desired release rate. These results indicate that ISFIs are a viable platform for the release of HAase and that the release rate is sufficient to maintain constant enzymatic activity levels.

## Discussion

Long-acting injectable implants offer an alternative drug delivery method that can provide sustained drug release directly at the local targeted site, rather than transient, systemic release. Local drug delivery with these implants may prove especially beneficial in applications where systemic drug delivery has limited efficacy, as is the case with PDAC. Systemic delivery of drug to the tumor site is dictated by both convective and diffusive mass transfer. However, in PDAC, both processes are greatly hindered. In mice, the IFP in a normal pancreas is 8 to 13 mmHg, but this IFP is elevated to 75 to 130 mmHg in pancreatic tumors, which is greater than the pressure in the arterioles and capillaries (13). Thus, the elevated IFP in tumors essentially blocks convective transport. Additionally, diffusive transport is greatly hindered by the viscous and tortuous nature of the dense stroma, which is especially high in HA (6). Degrading this HA, whether through enzymatic or nonenzymatic methods, in the tumors can potentially improve these mass transport properties by reducing IFP and decreasing the stromal density/viscosity. This should allow for any subsequent systemic chemotherapy to have improved mass transport into the tumor while the patency is restored and the viscosity is reduced. As a first step, this study focused on the development and characterization of an ISFI that could be used for sustained degradation of HA directly at the tumor site. Beyond affecting the distribution of molecular therapies, HA content can also affect immune cell infiltration. Recent studies have demonstrated that immune cells migrate more efficiently on soft tumors (with a 16 kPa shear modulus) vs. stiff tumors (with a 50 kPa shear modulus), which indicate that matrix modifying therapies have the potential to create a tumor microenvironment that is more amenable to both targeted therapies as well as immunotherapies (49).

We have shown that our 15-kDa implant does provide sustained HAase release over 21 d and that this HAase remains bioactive after release. The HAase from the implant retains its bioactivity not just in an in-vitro HA environment but also on human HA produced within a tumor and after injection into murine tumors. We can quantify the reduction in HA viscosity and molecular weight due to the implant as well as directly visualize the loss of the HA in the tumors. Of note is that the blank implant reduces the HA viscosity just as much as the HAase-ISFI by 14 d. It appears that acid-catalyzed degradation of HA becomes more significant than enzymatic degradation as the implant itself begins to degrade and release acidic monomers (lactic acid and glycolic acid) that significantly reduce the surrounding pH. Thus, our ISFI provides sustained degradation of HA, acting first through enzymatic degradation by releasing bioactive HAase and then through acid-catalyzed degradation as the implant degrades into acidic by-products.

This formulation holds promise as an intratumoral injection to degrade HA in pancreatic tumors. We have shown that the implant can degrade HA in the tumor microenvironment. We have also estimated that an enzyme release rate of 25.0 µg/d should be sufficient to maintain constant HAase activity levels such that sustained degradation of HA could be achieved. The 15 kDa implant does release HAase at a comparable rate of 11.1 µg/d. However, the implant properties can be further tuned in the future to optimize the mass release. For example, the molecular weight of the polymer can be tuned to increase the total mass of HAase released over time or a more purified HAase with a higher initial specific activity can be used to increase the potency of the HAase that is released. While our deactivation kinetics are based on in-vitro stability and do not include additional mechanisms of action that would be found in vivo, here we do show proof-of-concept that our ISFI system can release the amount of HAase necessary to provide for sustained degradation of HA.

## Materials and methods

### Implant formation and characterization

Polymer solution was created by combining poly(lactic-co-glycolic) acid (PLGA, LG 50:50, acid endcap, Mn 10 to 15 kDa), N-methyl-2-pyrrolidone (NMP), and hyaluronidase (HAase, bovine testicular, lyophilized powder, 685 USP units/mg) in a 39:60:1 mass ratio. A volume of 60 µL of this solution was injected into either 10 mL PBS (HAase release study) or 0.5 mL of a 10 mg/mL 1.5 MDa HA solution (characterization and functional testing) to form the implants. Implants were kept at 37°C on a shaker at 100 rpm. HAase release was quantified with a microBCA assay. Diffusion-weighted MRI was conducted and analyzed as previously described (48). See Supplemental Material for further details.

### Implant functional testing

Viscosity measurements were obtained using a TA Instruments AR-G2 rheometer on the 10 mg/mL HA solutions after injections. The primary human PDAC tissues slices were obtained from the Indiana University Cancer Center after having been formalin-fixed and paraffin-embedded. Serial slices were used from the same tumor. HA staining was done using a biotinylated hyaluronan binding protein and a streptavidin Qdot 705 secondary antibody. Lastly, KPC (Pdx1-Cre; LSL-Kras^G12D^; Tp53^R172H/+)^ cells were injected into mice subcutaneously as a xenograft model to form heterotopic pancreatic tumors used for ex-vivo injections and analysis. All animal studies were performed following protocols approved by the Purdue Animal Care and Use Committee. See Supplemental Material for further details.

## Supplementary Material

pgac193_Supplemental_Files

## Data Availability

All data are included in the manuscript and/or Supplimentary Material.
