# Supplementary material for: Sustained degradation of hyaluronic acid using an in situ forming implant
Source: PNAS Nexus. 2022 Sep 17;1(4):pgac193. doi: 10.1093/pnasnexus/pgac193 (PMC9802073; doi:10.1093/pnasnexus/pgac193)
Supplement: pgac193_Supplemental_Files [file pgac193_supplemental_files.zip › PNASNEXUS-PNASNEXUS-2022-00659-s01.docx]

**
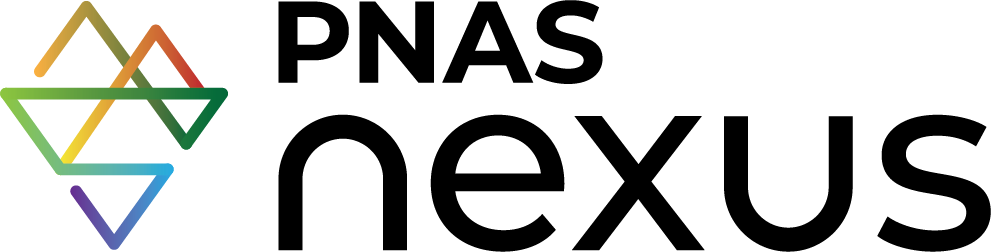
**

**Supplementary Information for**

Sustained Degradation of Hyaluronic Acid using an In Situ Forming Implant.

Kelsey Hopkins^1^, Kevin Buno^1^, Natalie Romick^1^, Antonio Carlos Freitas dos Santos^2,3^, Samantha Tinsley^4^, Elizabeth Wakelin^1^, Jacqueline Kennedy^1^, Michael Ladisch^2,3^, Brittany Allen-Petersen^4,5^, and Luis Solorio^1,5*^

Corresponding author: Dr. Luis Solorio

Email: [lsolorio@purdue.edu](mailto:lsolorio@purdue.edu)

**This PDF file includes:**

Supplementary Methods

**Other supplementary materials for this manuscript include the following:**

Supplemental Figures and Tables

**Supplementary Information Text**

**S1 Materials and Methods**

**S1.1 Materials**

All materials were used as received. Poly(lactic-co-glycolic) acid (PLGA, LG 50:50, acid endcap, Mn 10-15 kDa) was obtained from Akina PolySciTech. N-methyl-2-pyrrolidone (NMP) was obtained from Fisher Scientific. Hyaluronidase (HAase, bovine testicular, lyophilized powder, 685 USP units/mg) was obtained from Worthington Biochemical. 1.5 MDa sodium hyaluronate (HA) was obtained from Lifecore Biomedical. Sigmacote was obtained from Sigma-Aldrich. Phosphate-buffered saline (PBS) was obtained from Gibco. Slides of human pancreatic tumor specimens were obtained from the Indiana University Simon Comprehensive Cancer Center (Indianapolis, IN).

**S1.2 Formulation of Implant Polymer Solutions**

To form the polymer solution, PLGA, NMP, and HAase were combined in a 39:60:1 mass ratio. HAase powder was first suspended in NMP, then PLGA was added. Solutions were stirred overnight at room temperature to fully mix before transfer to 4ºC for storage.

To form blank implants as vehicle controls, no HAase was incorporated into the polymer solution. PLGA and NMP were combined in a 40:60 mass ratio, and solutions were stirred overnight.

**S1.3 Preparation of HA Solutions**

All HA solutions were prepared at 10 mg/mL in PBS. The 1.5 MDa HA powder was dissolved in the PBS by stirring at 4ºC for ≥ 1h. Solutions were then stored at 4ºC and used within 10 d.

**S1.4 Drug Release Study**

A 21 d release study was conducted on implants injected into 10 mL of PBS in 20 mL scintillation vials. Implants were formed by injecting 60 µL of polymer solution into PBS. Before injection, the scintillation vials were coated with Sigmacote to prevent protein adsorption. Samples were taken from the PBS bath solution at 0.25, 0.5, 1, 2, 4, 6 h after implant formation and then again at 1, 2, 3, 4, 5, 6, 7, 10, 14, 17, and 21 d. The bath solution was completely exchanged with fresh PBS at each of these seventeen timepoints to maintain sink conditions. Samples were stored in low protein binding tubes at -20°C until analyzed.

At 21 d, the implants were degraded in 5 mL of 0.9 M NaOH to determine residual drug mass. The degradation solution was then neutralized with 0.9 M HCl before analysis. The protein content of each sample was determined using the Micro BCA Protein Assay (Thermo Scientific 23235). A HAase standard curve in PBS was used to calculate the mass of HAase released in each sample in order to plot cumulative release over time.

**S1.5 Implant Characterization in HA**

The following characterization studies were carried out on implants formed by injecting 60 µL of HAase polymer solution into 0.5 mL of 10 mg/mL HA. The implants had an average mass of 36.0 ± 8.3 mg and were kept at 37ºC on a shaker at 100 rpm.

***Erosion and Solution Uptake***

The initial mass of each implant was recorded immediately after injection of the polymer solution. At each timepoint, implants were removed from the bath solution and their final, wet mass was recorded. The implants were then frozen at -80°C prior to lyophilization for two days. After lyophilization, the final, dry mass was recorded. The HA bath solution was replaced at 24 h due to its degradation.

Samples were taken at the following time points: 1, 3, 5, 7, 10, 14, 17, 21 d after implant formation.

Erosion was calculated by dividing the implant’s dry mass by its initial mass and normalizing it to the initial polymer mass fraction of 0.39:

$$normalized erosion= \frac{\frac{dry mass}{initial mass}}{initial polymer mass fraction}$$

Solution uptake was calculated using the wet mass of the implant as follows:

$$\% soln uptake= \frac{(wet mass-initial mass)}{initial mass}*100\%$$

***Scanning Electron Microscopy (SEM)***

To analyze implant microstructure, selected implants were imaged with SEM. After 1, 3, 7, 10, or 14 d in solution, implants were freeze-fractured over dry ice and then lyophilized for two days. Lyophilized implants were mounted on aluminum stubs and sputter-coated with 3-4 nm of platinum using a Cressington 208 HR sputter coater. A FEI NovaNanoSEM with a spot size of 3, a voltage of 5.00 kV, and a working distance of approximately 5 mm was used for imaging.

***Diffusion-weighted MRI (DWI)***

Diffusion-weighted imaging was conducted on implants as previously described [1]. Briefly, DWI was conducted using a Bruker BioSpec 70/30 USR 7T Preclinical MRI system and Bruker RF RES 300 1H 075/040 QSN TR rat head/mouse body volume coil. Implants at each timepoint were imaged using a standard diffusion-weighted spin echo protocol (TE=17.5 ms, TR=2500 ms, FOV=35x35 mm^2^, slice thickness=0.80 mm, b=0,1000 s/mm^2^). Timepoints were as follows: 1 h and then 1, 3, 5, 7, 10, 14 d after implant formation.

***DWI Analysis***

Analysis of the DWI data was as previously described [1]. Briefly, apparent diffusion coefficient (ADC) maps were created from the raw diffusion data using Matlab. The ADC maps had an image size of 128x128 pixels with a resolution of 0.273 mm/pixel. From the ADC maps, the implant was selected as the region of interest (ROI) by manual selection using a custom Matlab code. The mean diffusivity (MD) of the implant ROI was calculated as the average diffusion coefficient of the selected pixels. The diffusivity was also evaluated as a function of implant radius. The ROI was eroded away in layers one pixel width at a time. The MD of each eroded ring was calculated until the center of the implant was reached.

**S1.6 Hyaluronidase Functional Assays**

To assess the bioactivity of the HAase after release from the implant, the following functional assays were performed.

***HA Viscosity Measurements over Time***

HAase-ISFIs were formed in HA as described in Section S1.5. Control groups were injected for comparison. As a negative control, 60 µL of PBS was injected into 0.5 mL of 10 mg/mL HA, while 60 µL of 2.5 mg/mL HAase was injected as a positive control. Additionally, 60 µL of a blank (no-drug) implant was injected as a vehicle control. All solutions were kept at 37ºC on a shaker at 100 rpm.

At selected timepoints (1, 3, 5, 7, 10, 14 d), a 40 µL sample of the HA was removed from each tube for viscosity measurements. The pH of this HA bath solution was also measured at these same timepoints. Viscosity measurements were obtained using a TA Instruments AR-G2 rheometer. A 20 mm 1º steel cone was used as the test geometry. 40 µL of test solution was placed onto the plate. The cone was then lowered to a gap height of 29 µm. Plates were kept at 37 ºC and a humidified solvent trap cover was used to prevent dehydration. A 3 min time sweep with a 5% strain and frequency of 1 Hz was used to measure n’ (Pa s) of each sample. These parameters were chosen as they were determined to be within the linear viscoelastic region of the hydrogel. Measurements of n’ were taken every 10 s over the course of the 3 min time sweep, and the average n’ was calculated to determine the viscosity of each sample. The same viscosity measurements were also done on the pure 10 mg/mL HA solution to get an initial baseline viscosity used to normalize all measurements.

Due to the degradation of the HA solution after 24 h of incubation with the HAase-implant, the HA solutions for each implant were completely replaced with 0.5 mL of fresh 10 mg/mL HA after the 1 d timepoint was recorded. The implant then remained in this second HA solution for the remainder of the 14 d study. In addition, the positive control HAase injection had also degraded its HA solution after 24 h of incubation. Thus, there was no need for further viscosity measurements on the HAase control after the 1 d timepoint.

***Size-exclusion Chromatography (SEC) on HA***

At selected timepoints (1 d and 14 d), a sample of the HA bath solution used for the viscosity measurements was set aside for SEC measurements. HA samples were first diluted 10x in PBS (to 1 mg/mL) in order to have a concentration within the working range of the SEC column. Prior to SEC analysis, each sample was syringe-filtered through a 0.2 µm filter.

A Waters e2695 Separations Module (Waters Corporation, Milford, MA) was used to store samples at 4°C before injection. The injection volume was 20 μL with PBS buffer as the mobile phase at 1 mL/min flow rate. A two-column series PL aquagel-OH 60 (7.5 x 300 mm, 8 µm) and PL aquagel-OH 40 (7.5 x 300 mm, 8 µm), both acquired from Agilent Technologies (Santa Clara, CA), with a guard column was used for SEC. The columns were kept at room temperature. Polymers were detected using two inline detectors 2414 Refractive Index detector and 2489 UV detector (220 nm) (Waters Corporation, Milford, MA). Data was collected for 30 minutes. Molecular weight was determined using Empower 4 (Waters Corporation, Milford, MA), and the system was calibrated using InfinityLab EasiVial PEO/PEG Standards (Agilent Technologies, Santa Clara, CA).

***Patient Tumor Slide HA Staining***

Slides of primary human PDAC tissue were obtained from the Indiana University Simon Comprehensive Cancer Center. The tissues had been formalin-fixed and paraffin-embedded, and then 4 µm sections were mounted on slides. All four tumor slides used in this study were serial sections from the same tumor tissue. Four different groups were tested, one sample on each of the four tumor slides. The test group was the 1 d releasate sample from the HAase-ISFI release study (Section S1.4), which had a concentration of 2.88 µg/mL HAase in PBS. This 1 d release timepoint was used as it represents the end of the burst release phase and a high concentration of HAase was present as a result. A freshly-prepared HAase solution in PBS at the same concentration (2.88 µg/mL) was used as a positive control. A PBS solution was used as the negative control. Lastly, a secondary antibody control (with no primary antibody staining) was performed to confirm antibody specificity.

Prior to use, the tissue slides were deparaffinized by soaking in 100% xylene for five minutes, twice. Slides were then rehydrated in a series of three-minute rinses in an ethanol ladder: 100%, 100%, 95%, 70%, and 50%. The slides were then rinsed twice in PBS for five minutes. A hydrophobic barrier was created around the tissue on the slide using a PAP pen. Then, 300 µL of each of the four samples described above was incubated on the slides for two hours at 37°C before rinsing off with PBS.

To stain for HA, a biotinylated hyaluronan binding protein (HABP; Sigma Millipore 385911) was diluted 1:100 in 1% BSA in PBS, and 200 µL was added to each slide (excluding the secondary control) and allowed to sit overnight at 4°C. On the following day, samples were rinsed with PBS in the following manner: 3×5 minutes & 3×15 minutes. For secondary staining, streptavidin QDot 705 was attached to the biotinylated HABP. The streptavidin Qdot 705 was diluted to 1:200, and 200 µL was added to each slide and allowed to sit overnight at 4°C. Samples were again rinsed with PBS: 3×5 minutes & 3×15 minutes. Finally, 40 µL of antifade reagent with DAPI was added to the slide and a coverslip was placed onto the sample. The sample sat at room temperature for 15 minutes before placing into 4°C overnight. The next day, the coverslips were sealed, and the samples were imaged on a Zeiss LSM 800 confocal microscope.

Image analysis was performed on the confocal images to quantify the amount of HA staining (red) still present after treatment. The changes in HA were calculated by measuring the percent of red pixels present in the image. The total number of red pixels was obtained by setting a threshold based on histograms of the image. Any pixels in the red channel with an intensity > 55 were determined to be red. The number of red pixels in each image was then divided by the total number of pixels to obtain the percent red (or %HA) of the image.

***Injections into Ex Vivo Murine Tumors***

All animal studies were performed following protocols approved by the Purdue Animal Care and Use Committee. As proof of concept that our implants could be injected into a pancreatic tumor environment, *ex vivo* heterotopic pancreatic tumors from mice were used. Subcutaneous injections were prepared using a 1:1 mixture of serum free DMEM and Matrigel (Corning; CB-40234) as a cell suspension containing 500,000 KPC (Pdx1-Cre; LSL-Kras^G12D^; Tp53^R172H/+)^ cells per 50 µL injection volume. To form subcutaneous tumors, 100 µL injections were administered into wild-type C57BL/6 mice just below the dermal layer on both the left and right flanks of the mice (two injections per mouse). Mice were monitored as tumor growth occurred. At 17 d post-injection, mice were euthanized, and the subcutaneous tumors were harvested and fixed in 10% neutral buffered formalin.

***Alcian Blue Staining on Ex Vivo Murine Tumors***

HAase-ISFI polymer solution (made as in Section S1.2) was loaded into a 1 mL syringe and a 20 G needle was attached. The solution was then injected into the center of fixed *ex vivo* tumors described above. Control solutions were also injected, namely PBS as a negative control and 2.5 mg/mL HAase as a positive control. A blank implant solution was also injected. After injection, the tumors were incubated at 37°C for 2 d before staining. To control for the HA variability in these tumors, each of the four tumors was cut in half prior to injections. One half was used for the injection (HAase-ISFI, PBS, Blank-ISFI, or HAase solution), and the other was used as a control to quantify the amount of HA initially present in each tumor.

Histology was performed by the Histology Research Laboratory in the College of Veterinary Medicine at Purdue University. The tumors were paraffin-embedded, sectioned, and then Alcian Blue pH 2.5 staining was performed to analyze glycosaminoglycan (GAG) content. As another control, for each tumor, a serial section was treated with hyaluronidase prior to Alcian Blue pH 2.5 staining.

Image analysis was performed on the histology images to quantify the amount of HA staining (blue) still present after treatment. The ImageJ color deconvolution plugin [2] [3] using the built-in ‘Alcian Blue & Haematoxylin’ stain vector was used on the original histology images to separate out the Alcian Blue stain. Then, Matlab was used to quantify the number of blue pixels in the deconvoluted image by setting a threshold based off the hyaluronidase-treated serial section control images. The number of blue pixels in the tissue was divided by the total number of pixels in the tissue to get the percent blue within each tissue slice. By also doing this on the serial control sections that were treated with hyaluronidase before staining, the background non-specific GAG content that remained after hyaluronidase treatment could be subtracted out from the sample images so that the amount of HA in each image could be determined as follows:

$$\frac{\left( \text{\% blue in sample} \right) -\text{ (\% blue in haase-treated serial section of sample)}}{\left( \text{\% blue in no-inj ctrl} \right) -\text{ (\% blue in haase-treated serial section of no-inj ctrl)}}$$

**S1.7 Statistical Analysis**

Minitab 16.1.0 statistical software was used for all analyses. A one-way ANOVA with Tukey’s multiple comparisons at a confidence level of 95% was used to test for statistical significance between multi-group data. A 2-sample t-test at a confidence level of 95% was used to analyze two-group data. Resulting p-values less than 0.05 were considered significant. All values were reported as mean ± standard deviation.

**S2 Supplemental References**

| [1] | K. A. Hopkins, N. Vike, X. Li, J. Kennedy, E. Simmons, J. Rispoli and L. Solorio, “Noninvasive characterization of in situ forming implant diffusivity using diffusion-weighted MRI,” *Journal of Controlled Release,* vol. 309, pp. 289-301, 2019. |
| --- | --- |
| [2] | A. C. Ruifrok and D. A. Johnston, “Quantification of histochemical staining by color deconvolution,” *Anal. Quant. Cytol. Histol.,* vol. 23, pp. 291-299, 2001. |
| [3] | G. Landini, G. Martinelli and F. Piccinni, “Colour Deconvolution – stain unmixing in histological imaging.,” *Bioinformatics,* 2020. |
