# Supplementary material for: Sustained degradation of hyaluronic acid using an in situ forming implant
Source: PNAS Nexus. 2022 Sep 17;1(4):pgac193. doi: 10.1093/pnasnexus/pgac193 (PMC9802073; doi:10.1093/pnasnexus/pgac193)
Supplement: pgac193_Supplemental_Files [file pgac193_supplemental_files.zip › PNASNEXUS-PNASNEXUS-2022-00659-s02.docx]

**
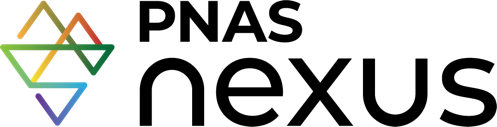
**

**Supplementary Information for**

Sustained Degradation of Hyaluronic Acid using an In Situ Forming Implant.

Kelsey Hopkins^1^, Kevin Buno^1^, Natalie Romick^1^, Antonio Carlos Freitas dos Santos^2,3^, Samantha Tinsley^4^, Elizabeth Wakelin^1^, Jacqueline Kennedy^1^, Michael Ladisch^2,3^, Brittany Allen-Petersen^4,5^, and Luis Solorio^1,5*^

Corresponding author: Dr. Luis Solorio

Email: [lsolorio@purdue.edu](mailto:lsolorio@purdue.edu)

**This PDF file includes:**

Figures S1 to S8

Tables S1 to S2

**Other supplementary materials for this manuscript include the following:**

Supplementary Methods


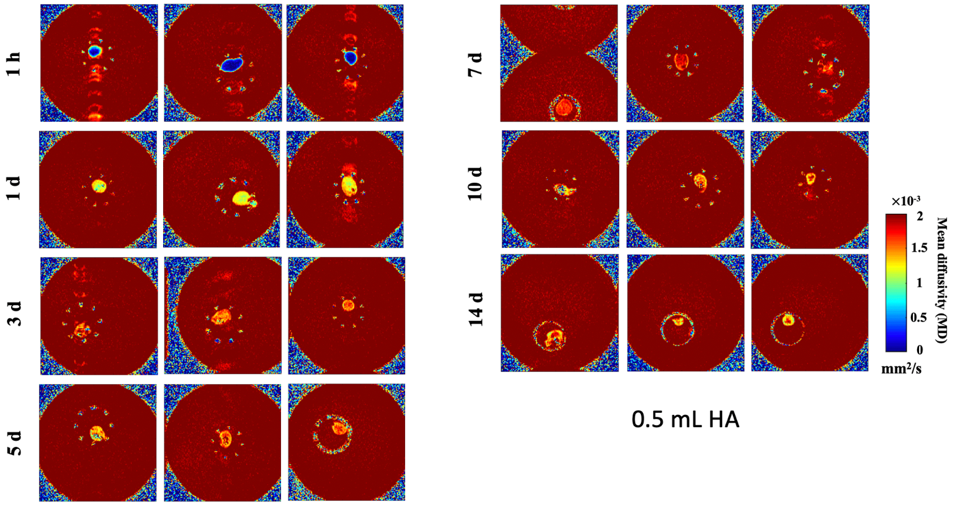
**Fig. S1.** Full set of apparent diffusion coefficient (ADC) maps for all 0.5 mL HA implants. Implants imaged in freely-diffusing water bath (red background).


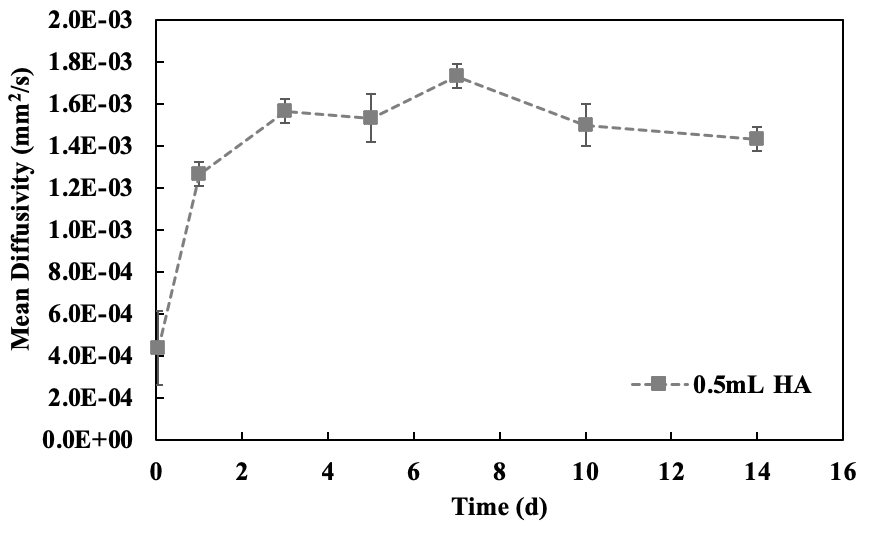
**Fig. Fig. S2.** Overall Mean Diffusivity (MD). Implant diffusivity from diffusion-weighted imaging. MD was calculated as the average diffusivity of the entire implant ROI at each timepoint.


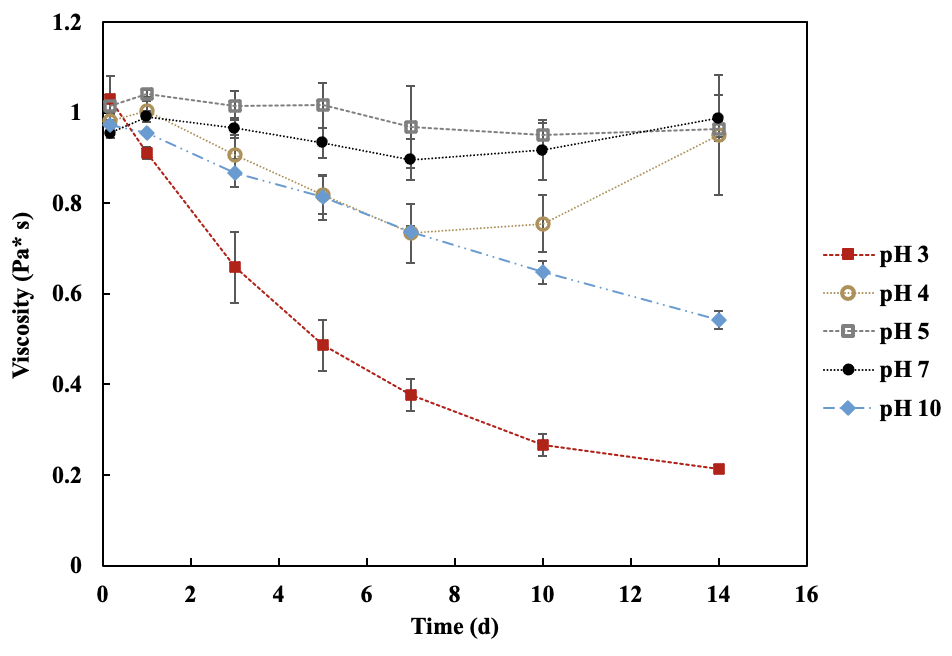
**Fig. Fig. S3.** Viscosity of a 10 mg/mL HA solution over 14 d normalized to its initial viscosity. The HA solutions were buffered at five different pH values over the course of the study. The pH of the HA solutions remained constant over time, while differences in viscosity were seen.


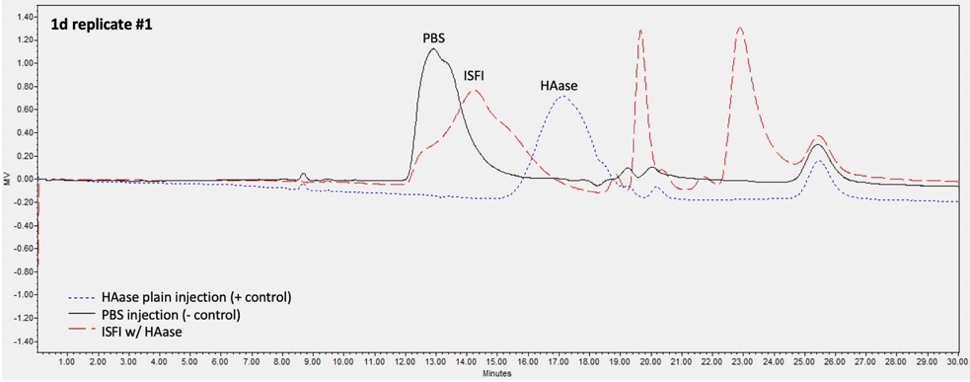


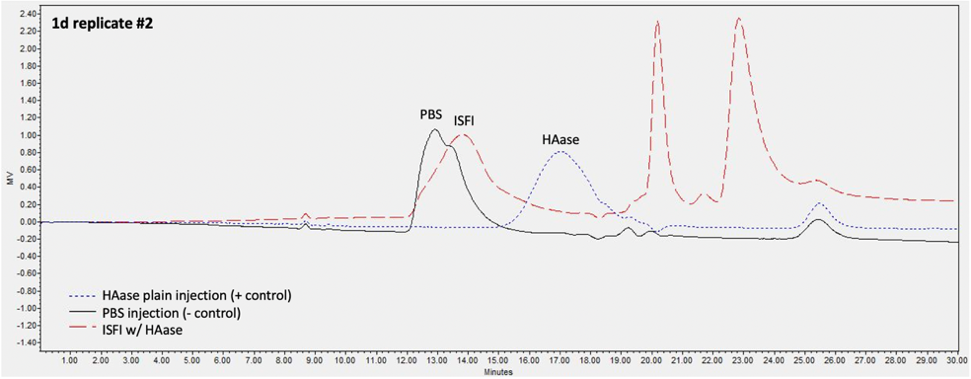


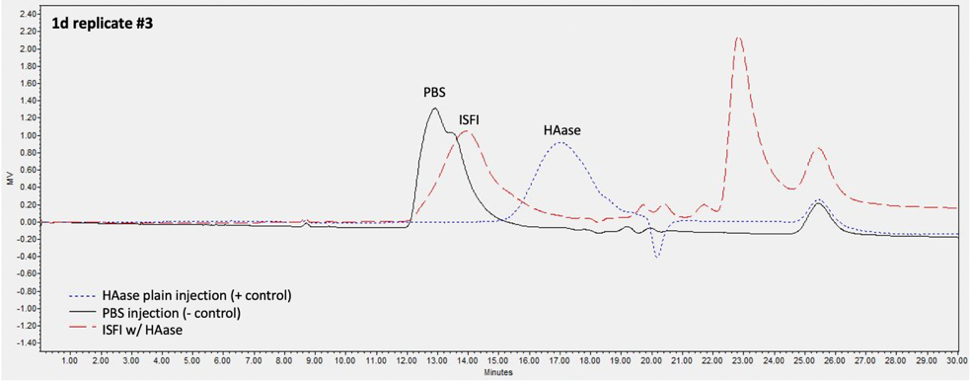


**Fig. S4.** Raw spectra from SEC analysis on 1.5 MDa HA injected with 2.5 mg/mL HAase, PBS, or HAase-releasing ISFI after 24 h. The molecular weight of the first peak was quantified.


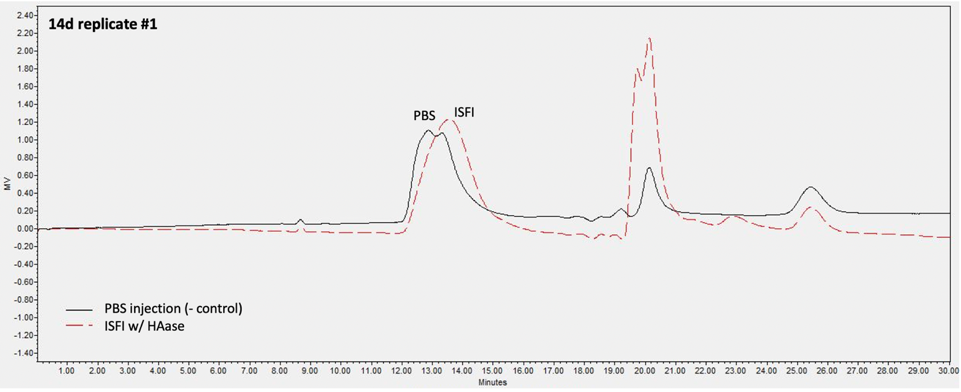


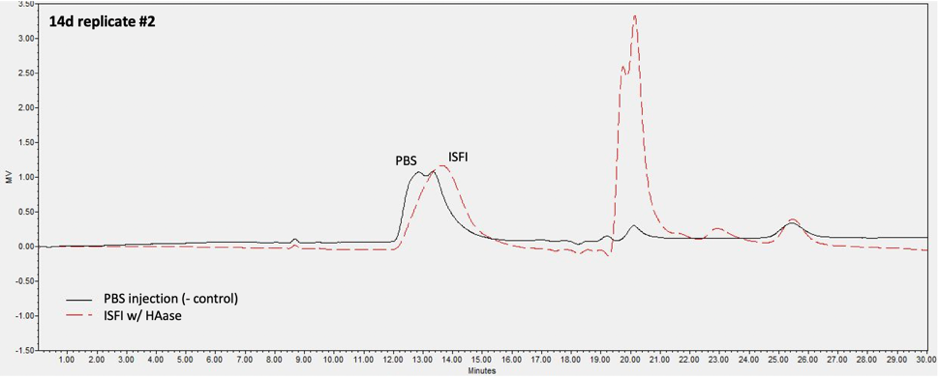


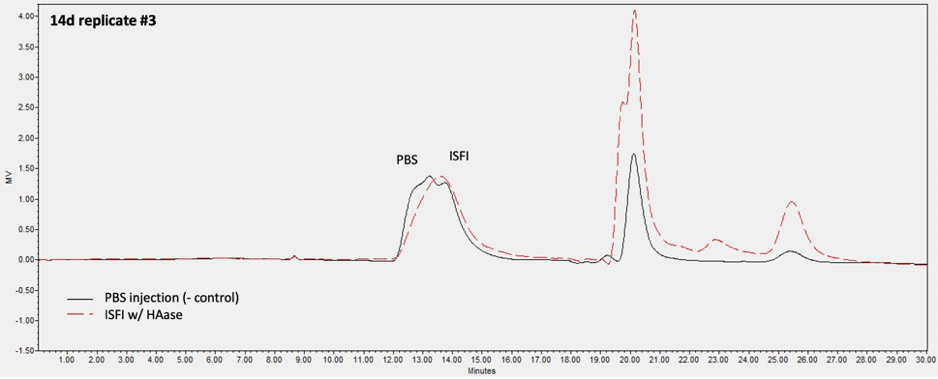


**Fig. S5.** Raw spectra from SEC analysis on 1.5 MDa HA injected with PBS or HAase-releasing ISFI for 14 d. The molecular weight of the first peak was quantified.

**
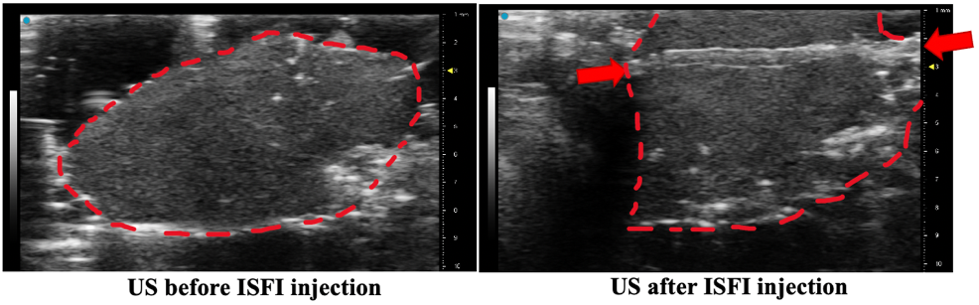
**

**Fig. S6.** Injection into ex vivo murine pancreatic tumors. Ultrasound (US) imaging showing the tumor (outlined in red) before and after injection of the implant, which can be seen as a straight cylindrical object across the entirety of the tumor cross-section between the red arrows.


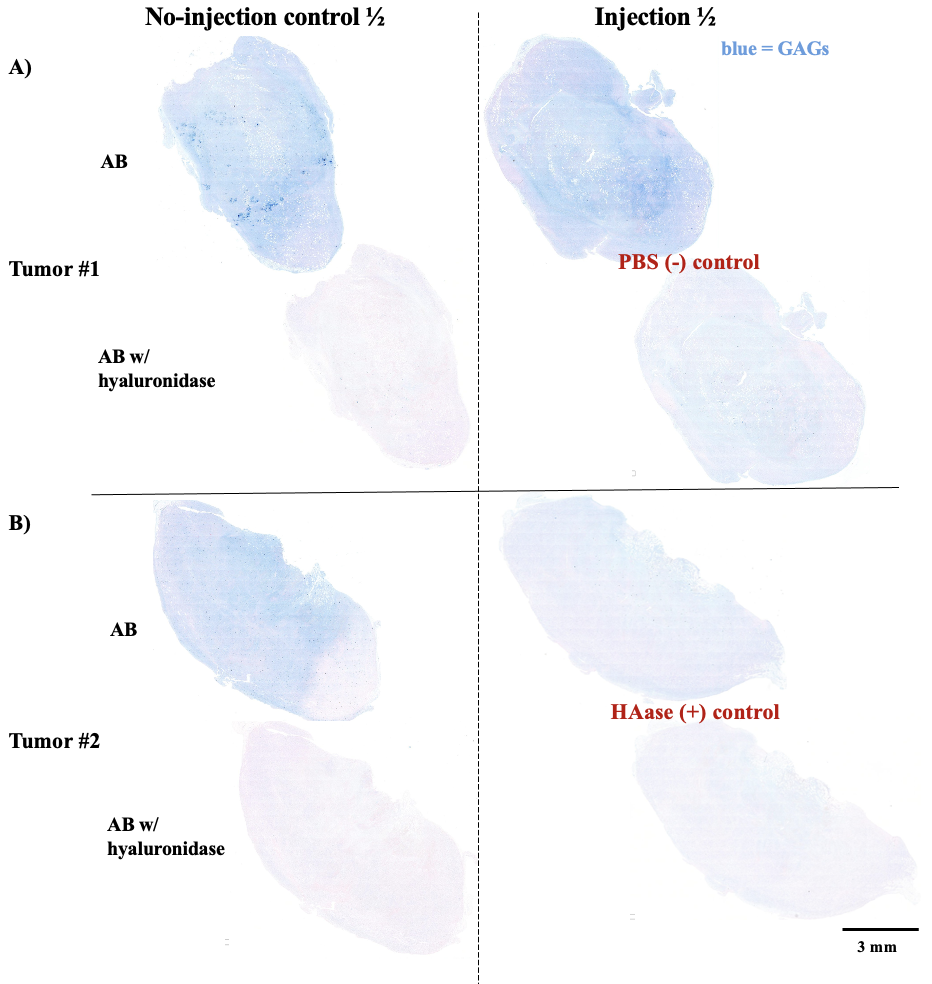


**Fig. S7.** Full set of images for Alcian blue pH 2.5 staining after injection into ex vivo murine subcutaneous pancreatic tumors. For each tumor, one half not touched and left as a no-injection control and the other half was subjected to the following injections **A,E,I)** PBS; **B,F,J)** 2.5 mg/mL HAase solution; **C,G,K)** HAase-releasing ISFI; and **D,H,L)** Blank implant. This supplemental figure also shows the serial slice that was treated with hyaluronidase before alcian blue staining. Continued on next pages.


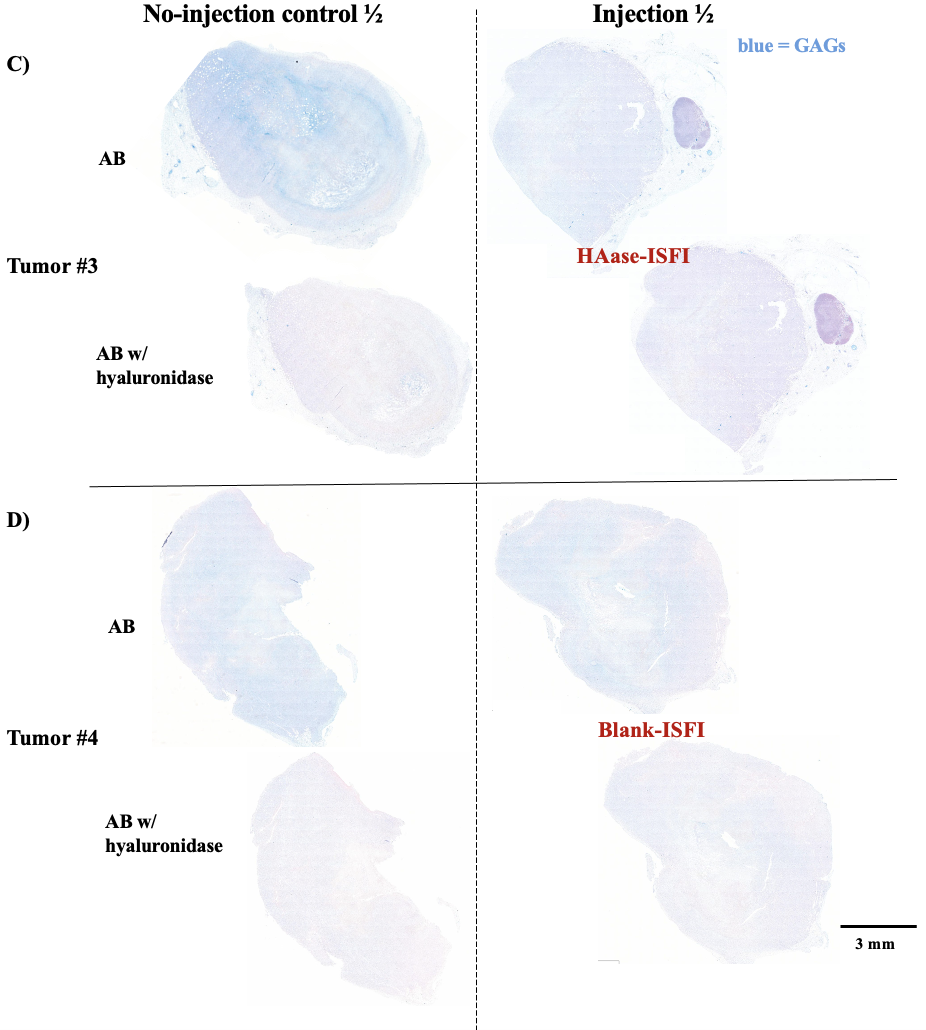


**Fig. S7.** Full set of images for Alcian blue pH 2.5 staining after injection into ex vivo murine subcutaneous pancreatic tumors. For each tumor, one half not touched and left as a no-injection control and the other half was subjected to the following injections **A,E,I)** PBS; **B,F,J)** 2.5 mg/mL HAase solution; **C,G,K)** HAase-releasing ISFI; and **D,H,L)** Blank implant. This supplemental figure also shows the serial slice that was treated with hyaluronidase before alcian blue staining. Continued on next pages.


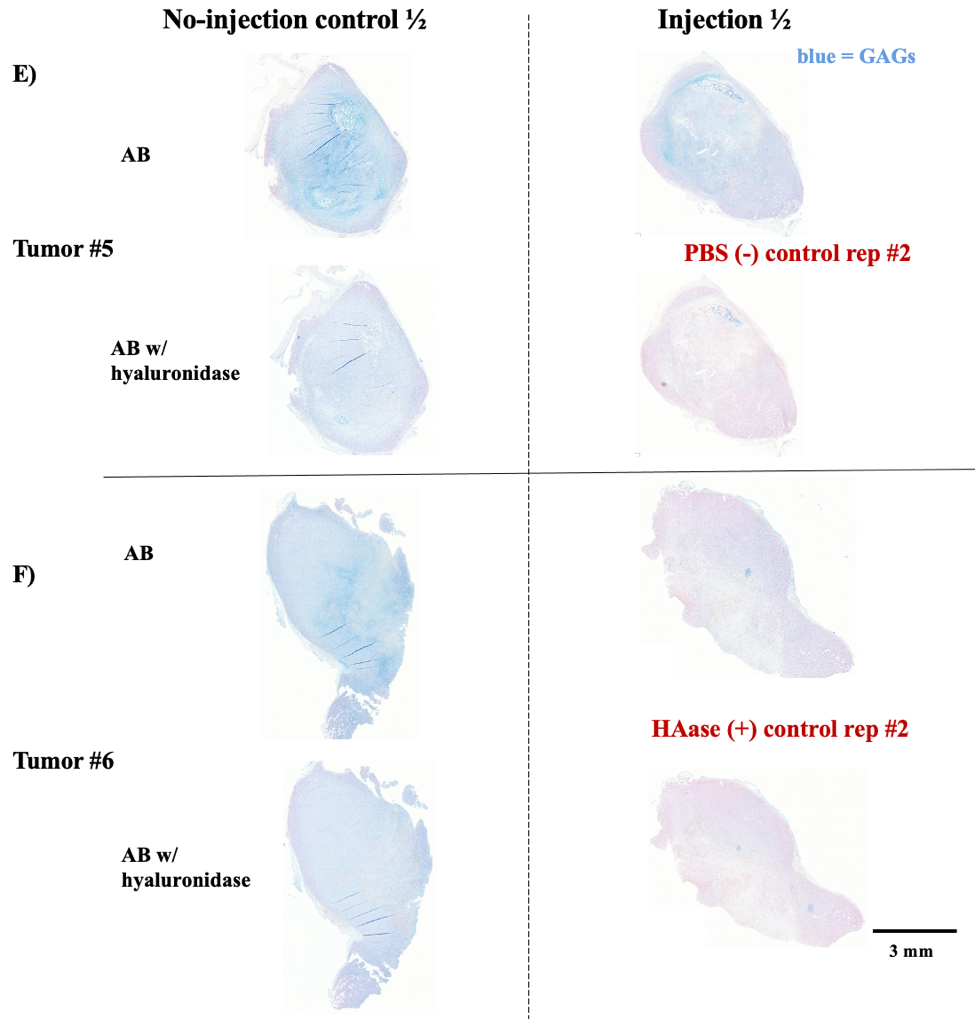


**Fig. S7.** Full set of images for Alcian blue pH 2.5 staining after injection into ex vivo murine subcutaneous pancreatic tumors. For each tumor, one half not touched and left as a no-injection control and the other half was subjected to the following injections **A,E,I)** PBS; **B,F,J)** 2.5 mg/mL HAase solution; **C,G,K)** HAase-releasing ISFI; and **D,H,L)** Blank implant. This supplemental figure also shows the serial slice that was treated with hyaluronidase before alcian blue staining. Continued on next pages.


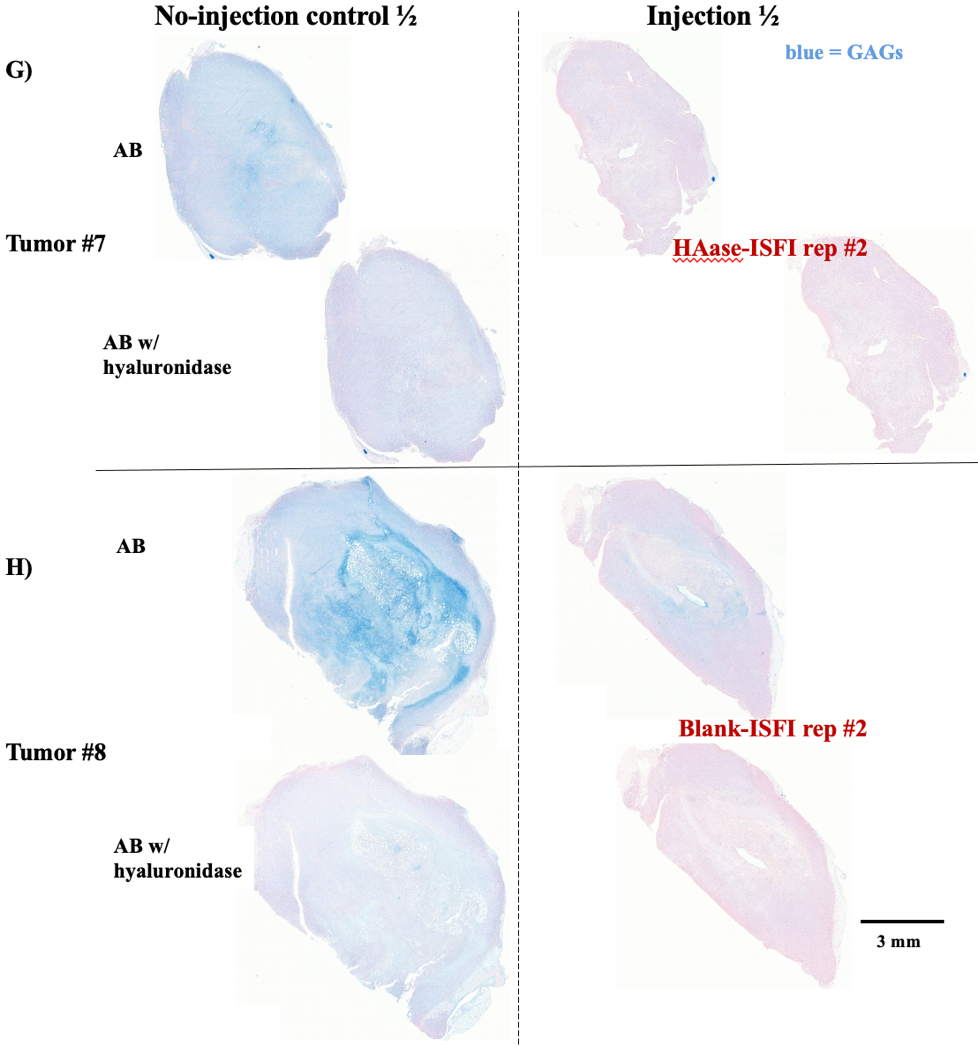


**Fig. S7.** Full set of images for Alcian blue pH 2.5 staining after injection into ex vivo murine subcutaneous pancreatic tumors. For each tumor, one half not touched and left as a no-injection control and the other half was subjected to the following injections **A,E,I)** PBS; **B,F,J)** 2.5 mg/mL HAase solution; **C,G,K)** HAase-releasing ISFI; and **D,H,L)** Blank implant. This supplemental figure also shows the serial slice that was treated with hyaluronidase before alcian blue staining. Continued on next pages.


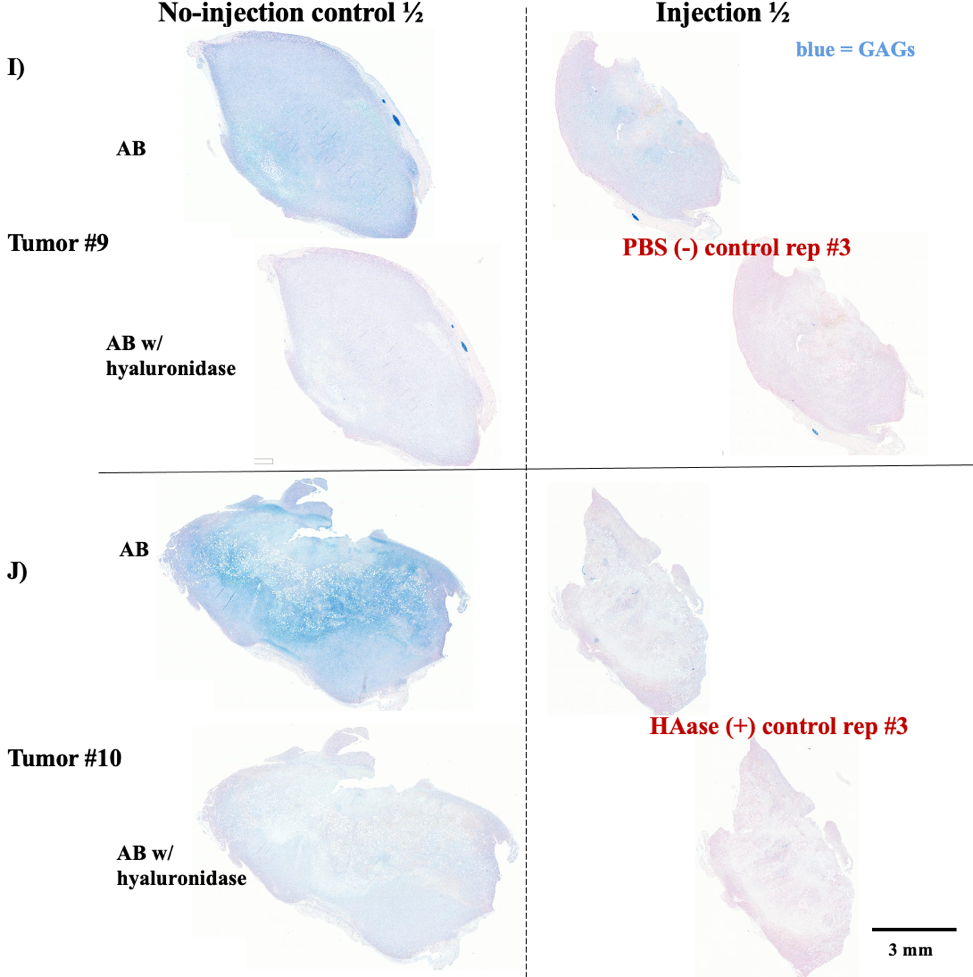


**Fig. S7.** Full set of images for Alcian blue pH 2.5 staining after injection into ex vivo murine subcutaneous pancreatic tumors. For each tumor, one half not touched and left as a no-injection control and the other half was subjected to the following injections **A,E,I)** PBS; **B,F,J)** 2.5 mg/mL HAase solution; **C,G,K)** HAase-releasing ISFI; and **D,H,L)** Blank implant. This supplemental figure also shows the serial slice that was treated with hyaluronidase before alcian blue staining. Continued on next page.


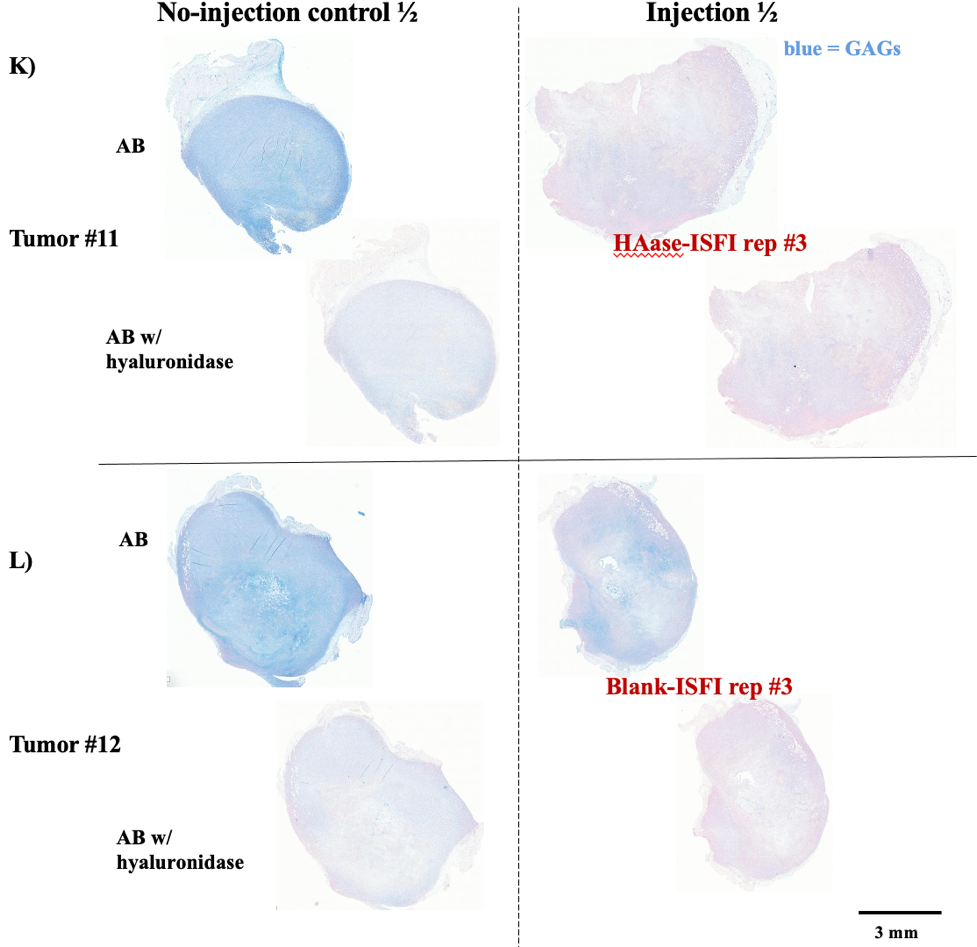


**Fig. S7.** Full set of images for Alcian blue pH 2.5 staining after injection into ex vivo murine subcutaneous pancreatic tumors. For each tumor, one half not touched and left as a no-injection control and the other half was subjected to the following injections **A,E,I)** PBS; **B,F,J)** 2.5 mg/mL HAase solution; **C,G,K)** HAase-releasing ISFI; and **D,H,L)** Blank implant. This supplemental figure also shows the serial slice that was treated with hyaluronidase before alcian blue staining.

**Table S1.** Activity of 25 ug/mL HAase stored at 37°C in PBS over time with fit to first-order deactivation.


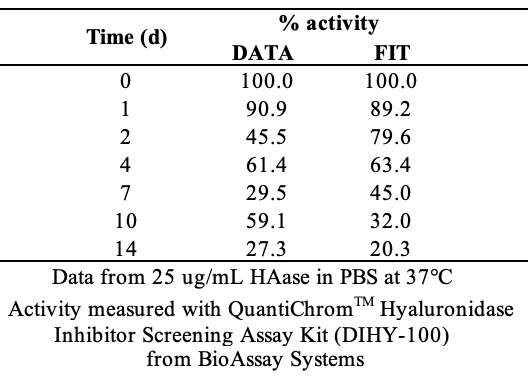


**Fig. S8.** Fit of first-order deactivation kinetics to HAase activity in PBS at 37°C, graphical version of Table S1.

**Table S2.** Parameters for HAase enzyme kinetics calculations.

**
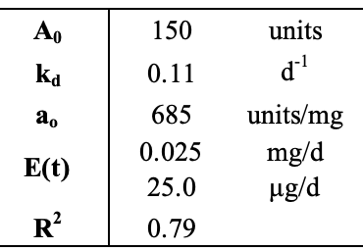
**

**Supplemental Text for Enzyme Kinetics**

First-order deactivation:

A(t) = ${A_{0}e}^{{-k}_{d}t}$ (eqn 1)

Mass flow rate to maintain constant activity:

E(t) = $\frac{A_{0}}{a_{0}}k_{d}$ (eqn 2)

Solve for $A_{0}$ and $k_{d}$ by fitting raw activity curve data (Table S1) to eqn 1 (Figure S8)

$a_{0}$ is initial specific activity associated with each batch of HAase

So here we have:

E(t) = $\frac{150 units}{685 units/mg}0.11 1/d$ = 0.025 mg/d or 25.0 µg/d

25 µg/d needed to maintain an activity level of 150 units

*(baseline estimation from in vitro data, not including in vivo mechanism of deactivation*
